# Disease burden of age-related macular degeneration in China from 1990 to 2019: findings from the global burden of disease study

**DOI:** 10.7189/jogh.11.08009

**Published:** 2021-10-30

**Authors:** Yichi Zhang, Aiming Chen, Minjie Zou, Zhenlan Yang, Danying Zheng, Min Fan, Guangming Jin

**Affiliations:** 1 Department of Ophthalmology, Sun Yat-sen Memorial Hospital, Sun Yat-sen University, Guangzhou, China; 2 Department of Pharmacy, Fifth Affiliated Hospital, Sun Yat-sen University, Zhuhai, China; 3 State Key Laboratory of Ophthalmology, Zhongshan Ophthalmic Center, Sun Yat-sen University, Guangzhou, China; 4 Department of General Intensive Care Unit, The Third Affiliated Hospital of Sun Yat-sen University, Guangzhou, China

## Abstract

**Background:**

To evaluate the disease burden of age–related macular degeneration (AMD) in terms of disability-adjusted life years (DALY) in China from 1990 to 2019.

**Methods:**

Prevalence of blindness and vision loss due to AMD and DALY number, rate, and age-standardized rates of AMD were collected from the Global Burden of Disease Study 2019 database. The characters of variables were analyzed between China and its neighboring countries.

**Results:**

From 1990 to 2019, the all-age number and rate for AMD prevalence and DALYs increased significantly in China, while the age standardized DALYs rate in 2019 showed a decrease of 3.63% compared with that in 1990. Females were found to have a higher prevalence and DALYs than males. The 65-69 age group had the highest AMD DALYs number, while the DALYs rate showed a positive association with age. In 2019, when compared to neighboring countries, the age standardized prevalence rate of AMD in China was ranked second after Pakistan, while the age standardized DALYs rate ranked second after Pakistan and India.

**Conclusions:**

Despite a small decrease in age standardized DALYs rate in China in the past three decades, the disease burden of AMD is still considerable and much higher compared to neighboring developed countries. Optimizing health services allocation is needed to further reduce this burden.

Age–related macular degeneration (AMD) is a degenerative disease of the macula; it has been one of the leading causes of severe and irreversible loss of vision globally [1-4]. The natural progression of AMD can be divided into early stage and advanced stage. Early AMD is characterized by the presence of soft drusen and/or pigmentary changes in macular. Some early cases can develop into late-stage AMD which includes two types: geographic atrophy and neovascular (exudative) AMD. Compared with early AMD, late AMD can cause irreversible loss of vision [3]. Although AMD is not a life-threatening disease, up to one–third of affected individuals will experience various degrees of disability and depression during the course of the disease, even when only one eye is affected [5,6]. In this way, AMD is a blinding disease that mostly targets the elderly [7]. Considering the growth of ageing populations, extended life expectancy worldwide, and falling death rates in most countries and territories, the prevalence and disease burden of AMD is likely to become a greater public health concern in the coming decades [8]. With China having the largest population in the world with a growing aging population, [9] it was estimated that China had the greatest number of people with AMD in 2019.

In recent years, an increasing number of epidemiological studies of AMD have been conducted [10-14], most of which focused on the incidence and distribution of AMD. The estimates were, however, contingent upon the characteristics of individual studies: the age structure of the study sample, case definition, geographic factors and classification of AMD. Furthermore, there were few studies focusing on the disease burden of AMD in China. Disease burden is typically measured by the disability-adjusted life years (DALY), which can quantify the healthy cost caused by the disease compared to the traditional epidemiological indicators. It is, therefore, important to have an updated investigation of the prevalence and disease burden of AMD in China. In this study, we extracted data from Global Burden of Disease (GBD) 2019 study which provided the AMD prevalence and disease burden in China and six neighboring countries from 1990 to 2019. These findings can contribute to providing critical information to stakeholders and guide health policies and health service approaches in China.

## METHODS

AMD prevalence and disease burden were extracted from the GBD 2019 study in the Global Health Data Exchange (GHDx, http://ghdx.healthdata.org/gbd-results-tool). Disease burden was presented as DALY. Details of the methodology of the study have been described in a previous study [8]. Briefly, DALYs attributable to AMD were defined as the sum of the years lived with disability (YLD) and the years of life lost (YLL), and was calculated by the following formula: DALY number = (Number of deaths × Standard life expectancy at age of death in years) + (Number of prevalent cases × Disability weight). The number of AMD DALYs was obtained, and Uncertainty Intervals (UIs) were defined as the 2.5th and 97.5th values of the ordered draw. The DALY rate was calculated by adjusting for population size as the number of cases per 100 000 population, while the age-standardized DALY rate was further adjusted for age structure.

The following data was obtained for further analyses: (1) gender-specific numbers, rates and age-standardized rates of prevalence and DALYs in China from 1990 to 2019; (2) Age-standardized rate prevalence and DALYs of seven neighboring countries of China (Japan, North Korea, South Korea, India, Singapore, Pakistan and Russia) in 1990 and 2019; (3) Numbers and age-standardized rates of prevalence and DALYs in main GBD regions (Global, High-income Asia Pacific, High-income North America, Western Europe, Australasia, Andean Latin America, Tropical Latin America) and different World Bank Income Level regions (World Bank High Income, World Bank Upper Middle Income, World Bank Lower Middle Income and World Bank Low Income) from 1990 to 2019. Outcomes including the time trends of AMD prevalence and DALYs from 1990 and 2019 in countries and territories mentioned above, the change rate of age-standardized rates prevalence and DALYs from 1990 and 2019 were further calculated. The Wilcoxon signed-rank test was used to assess differences in numbers and rate of DALYs between males and females in 2019. One-way ANOVA was used in the comparison of age-standardized prevalence and DALYs in China and the 7 neighboring countries in 1990 and 2019, and the Bonferroni correction was used for multiple comparisons. A P value <0.05 was considered statistically significant. Figures were drawn using GraphPad Prism software (version 5.01, GraphPad Prism Software; San Diego, CA, USA).

## RESULTS

### Time trends of AMD prevalence and DALYs from 1990 to 2019

**Figure 1:**
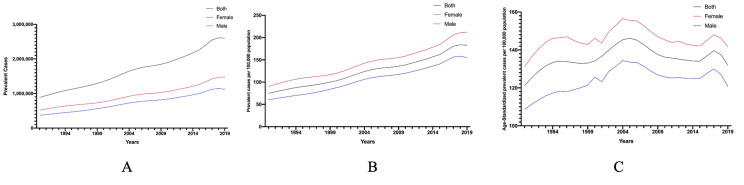
Prevalence of age-related macular degeneration from 1990 to 2019 in China.

**Figure 2:**
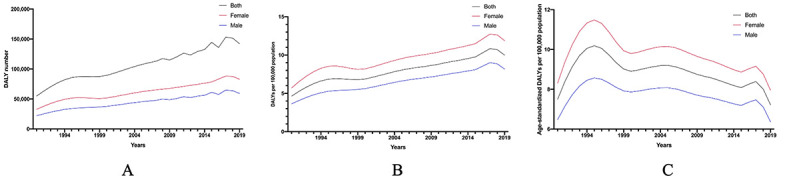
Disability-adjusted life years of age-related macular degeneration from 1990 to 2019 in China.

As **Figure 1** shows, the prevalent cases soared by 293.19% from 1990 (0.8 × 10^6^, 95% UI = 0.72 × 10^6^, 1.07 × 10^6^) to 2019 (2.6 × 10^6^, 95% UI = 2.13 × 10^6^, 3.11 × 10^6^), and the prevalent cases of females were more than males. Similarly, prevalence rate had an upward trend, with a 244.00% rise in the past 30 years (1990: 74.97, 95% UI = 60.94, 90.05; 2019: 182.93, 95% UI = 150.03, 218.99 per 100 000 population). However, after adjusting for population size and age structure, the age-standardized rate was 121.33 (95% UI = 100.83, 144.26) per 100 000 population in 1990, reached a peak in 2004 (146.21, 95% UI = 122.42, 171.70 per 100 000 population) and went down with a prevalence rate of 131.97 (95% CI = 109.37, 156.91) per 100 000 population in 2019. Prevalence of AMD in females surpassed those of males in prevalence number, prevalence rate and age-standardized prevalence rate from 1990 to 2019. Similar to the prevalence of blindness and vision loss, the DALYs number increased by 258.84% in the past 30 years, as it was 54 885.30 (95% UI = 37 530.28, 77 765.66) in 2019 compared to 142 065.75 (95% UI = 97 100.02, 197 277.11) in 1990 (**Figure 2**). The DALYs rate attributable to AMD trended upward (1990: 4.63, 95% UI = 3.17, 6.57; 2019: 9.99, 95% UI = 6.82, 13.86 per 100 000 population), while the age-standardized rate also reached its peak in the mid-1990s (1994: 10.18, 95% UI = 6.93, 14.38 per 100 000 population), finally dropping to 7.22 (95% UI = 4.91, 10.06 per 100 000 population), which decreased 3.63% from 1990 (7.49, 95% UI = 5.16, 10.54). DALYs of females were higher than males, but shared similar figures for prevalence of blindness and vision loss.

### Age and gender-specific disease burden attributable to AMD in 2019 in China

**Figure 3:**
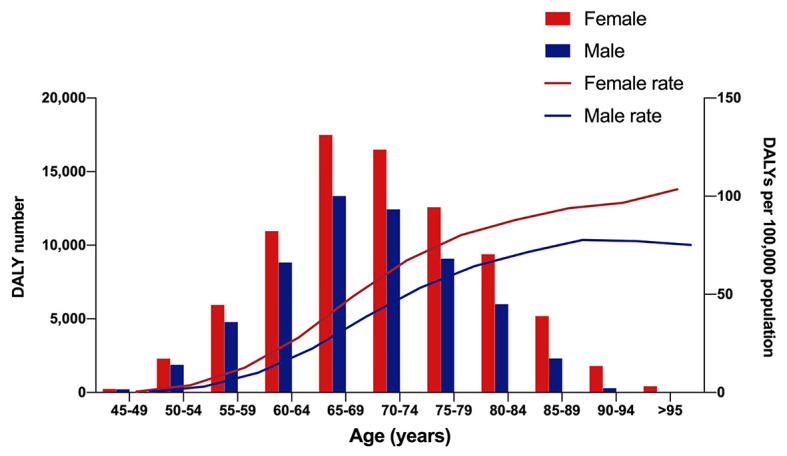
Age-related macular degeneration disability-adjusted life years in different age groups and rate in 2019.

**Figure 3** gives a brief view of age and gender-specific disease burden of AMD among the Chinese population. The AMD disease burden mainly fell on individuals aged 50 to 90 years, and the 65-69 age group were the most affected (Female: 17 488.47; Male: 13 346.17). The female DALYs rate climbed slightly with increasing age, while the male DALYs rate climbed slightly before the age of 90 and then decreased. Notably, females have higher DALYs attributable to AMD compared to males throughout each age group both in DALYs number and DALYs rate.

### Comparison of AMD burden between China and other regions

**Figure 4:**
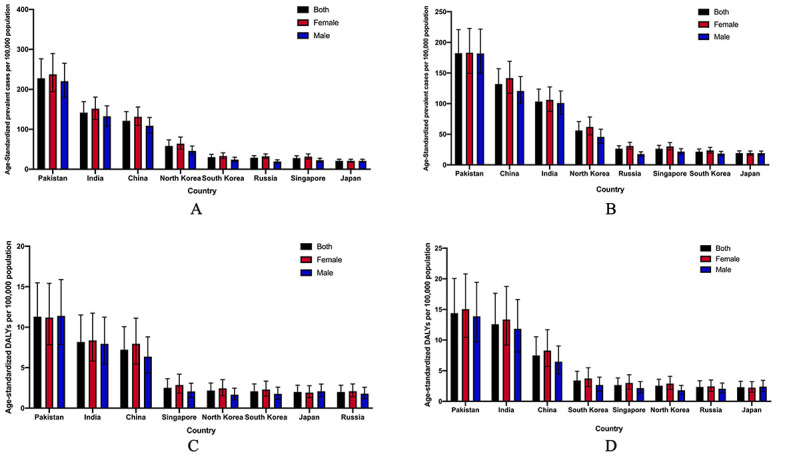
Distribution of age-standardized prevalence and disability-adjusted life years (DALY) due to age-related macular degeneration in China and other seven neighboring countries in 1990 and 2019. **A.** Age-standardized prevalence rate in 1990, **B.** age-standardized prevalence rate in 2019, **C.** age-standardized DALY rates in 1990 and **D.** age-standardized DALY rates in 2019.

Age-standardized prevalence of blindness and vision loss due to AMD and age-standardized DALYs caused by AMD in 1990 and 2019 were used as a basis for understanding the disease burden in China and 7 neighboring countries (**Figure 4**). China ranked third both in prevalence of blindness and vision loss due to AMD and age-standardized DALYs caused by AMD in 1990. In 2019, China rose to the second rank in the prevalence of blindness and vision loss due to AMD while DALYs rate were still ranked 3rd. Pakistan ranked first in prevalence of blindness and vision loss due to AMD and DALYs caused by glaucoma both in 1990 and 2019. Japan had the least prevalence of blindness and vision loss due to AMD and DALYs caused by AMD in 2019. Pakistan, India and China had a much higher prevalence of blindness and vision loss due to AMD and disease burden caused by AMD than the other 5 countries.

After adjusting for population and age, AMD disease burden in China was higher compared with most regions worldwide (**Table 1**). However, regions like World Bank Lower Middle Income and World Bank Low Income have an even higher disease burden than China. In terms of the percentage change in age-standardized rates from 1990 to 2019, unlike the increasing trend in prevalence of blindness and vision loss due to AMD (increased by 8.77%, 95% UI = 8.27%, 9.29%), the age-standardized DALYs rates caused by AMD decreased in China (decreased by -3.40%, 95% UI = -4.91%, -2.25%). The regions of World Bank Low Income, on the other hand, showed an increasing age standardized DALYs rates.

**Table 1:** Prevalent cases and DALY in 2019 for age-related macular degeneration in China for both sexes and percentage change of age-standardized rates by GBD regions

Countries or regions	Prevalent cases (95% UI)			DALYs (95% UI)		
**Counts (2019)**	**Age-standardized rates (2019)**	**Percentage change in age-standardized rates between 1990 and 2019 (%)**	**Counts (2019)**	**Age-standardized rates (2019)**	**Percentage change in age-standardized rates between 1990 and 2019 (%)**
China	2 601 883.03 (2 134 011.4, -3 114 761.87)	131.97 (109.37, 156.91)	8.77 (8.27, 9.29)	142 065.76 (97 100.02, 197 277.11)	7.22 (4.91, 10.06)	-3.40 (-4.91, -2.25)
Global	7 792 530.04 (6 526 081.5, 9 159 394.94)	96.76 (81.32, 113.20)	-2.04 (-2.34, -1.77)	564 055.10 (392 930.70, 789 194.64)	7.05 (4.92, 9.84)	-14.96 (-17.57, -12.60)
High-Income Asia Pacific	105 854.63 (87 551.31, 125 058.63)	19.69 (16.30, 23.36)	-11.39 (-12.78, -10.09)	10 622.68 (7 186.98, 15 401.48)	2.01 (1.36, 2.90)	-17.62 (-22.99, -13.06)
High-Income North America	187 099.23 (156 483.37, 219 524.61)	27.17 (22.58, 31.92)	-5.17 (-6.04, -4.38)	18 170.68 (12 528.56, 25 683.35)	2.64 (1.81, 3.75)	-9.28 (-13.21, -6.20)
Western Europe	976 388.69 (825 781.48, 1 138 807.18)	93.13 (78.71, 108.25)	-13.65 (-14.31, -13.01)	97 364.81 (67 087.70, 137 766.58)	9.31 (6.39, 13.09)	-19.18 (-21.58, -16.95)
Australasia	20 593.54 (17 012.15, 24 503.01)	37.96 (31.26, 45.24)	-11.56 (-12.55, -10.61)	2 080.78 (1 405.74, 2989.83)	3.86 (2.59, 5.58)	-17.17 (-20.90, -13.85)
Andean Latin America	61 461.71 (50 861.49, 73 157.27)	113.58 (94.10, 135.01)	-2.31 (-2.60, -2.05)	4 391.82 (3 022.64, 6180.43)	8.12 (5.58, 11.41)	-11.55 (-13.79, -9.55)
Tropical Latin America	193 602.30 (161 055.55, 230 808.98)	82.06 (68.01, 97.85)	-2.04 (-2.37, -1.75)	11 324.86 (7 848.25, 15 618.62)	4.82 (3.34, 6.77)	-5.86 (-8.26, -3.99)
World Bank High Income	1 442 595.81 (1 222 029.06, 1 679 476.12)	56.50 (47.67, 65.83)	-17.05 (-17.96, -16.16)	138 734.49 (96 224.71, 195 762.36)	5.43 (3.72, 7.60)	-22.87 (-26.15, -19.81)
World Bank Upper Middle Income	3 643 719.82 (301 800.26, 4 346 608.00)	109.78 (91.44, 130.09)	10.64 (10.04, 11.27)	216 026.12 (147 276.16, 300 471.34)	6.53 (4.48, 9.14)	-2.97 (-4.55, -1.82)
World Bank Lower Middle Income	2 361 108.82 (1 943 071.48, 2 823 342.44)	109.04 (90.55, 129.12)	17.14 (16.50, 17.79)	183 581.24 (126 110.39, 258 121.96)	8.53 (5.90, 11.98)	-23.84 (-26.45, -21.37)
World Bank Low Income	342 354.9 (284 927.70, 410 092.38)	127.63 (105.98, 150.92)	13.11 (12.49, 13.74)	25 470.66 (17 345.89, 35 366.04)	9.51 (6.50, 13.41)	0.74 (0.29, 1.52)

DALY = disability-adjusted life years, UI – uncertainty interval

## DISCUSSION

In this study, we reported the disease burden of AMD by year, age and gender in China from 1990 to 2019 by analyzing the annual follow-up data from the GBD study. Our findings could contribute to assisting policymakers in assessing and optimizing current health services for eye health. We found that the disease burden of AMD increased significantly from 1990 to 2019 and the current disease burden of AMD remains high, especially for females as well as the elderly. In addition, the AMD burden in China was significantly higher than neighboring developed countries, but similar to other middle-income neighboring countries.

Similar to our results, Xu et al [15] also reported gender disparity in AMD disease burden, with females being more heavily affected. This study also suggested that women were found to have higher DALYs than men across all age groups in China. This may be due to the following reasons: first, females may have a higher risk of developing neovascular AMD, which may severely affect sight [16]. Second, a longer life expectancy and a higher female proportion among the elderly population could be a possible explanation for this outcome [17]. Future strategies on improving eyecare services should prioritize reaching these higher risk populations in order to achieve a more targeted and cost-effective approach.

This study also demonstrated the prevalence of blindness and vision loss due to AMD and DALY rate of AMD approximately doubled in the past 30 years. Furthermore, the age-standardized prevalence rate of blindness and vision loss due to AMD in 2019 increased nearly 3 times compared to 1990. This could largely be due to the increasing and ageing population in China. Interestingly, when adjusted for population and age, the AMD disease burden showed a slight decrease in recent years in China. This may be due to a few reasons. First, the wide administration of anti-VEGF agents [18,19]. The emergence of anti-VEGF agents has revolutionized the treatment strategies against neovascular AMD, which can dramatically improve and prolong visual function, leading to alleviation of the AMD global burden. Second,the availability of new diagnostic tools such as optical coherence tomography (OCT) [20-22], fluorescein fundus angiography (FFA) and indocyanine green angiography [20], allow for the early detection and intervention of neovascular AMD. Third, progress in other therapeutic techniques (such as photodynamic therapy) also contribute to AMD therapy.

Meanwhile, we found that the burden of AMD mainly fell on people aged 60 to 85 years, with the 65-69 age group being most affected in both sexes; this may have been influenced by the relatively large number of patients in this age range. After adjusting for population size, the DALYs rate was positively correlated with age, and this is consistent with previous reports that age is a risk factor of AMD prevalence. Our results suggest that to alleviate the AMD disease burden, a more thorough screening protocol should be utilized for patients aged 60 to 85 years, especially for those aged 65 to 69.

The World Bank divides regions into high income regions, upper middle-income regions, lower middle-income regions, and low-income regions by income level. The AMD age standardized DALYs rate in 2019 in the World Bank high income regions, upper middle-income regions and lower middle-income regions were lower compared to DALYs rate in 1990, while the low-income regions showed an increasing trend in DALYs rate of AMD compared to 1990. The age-standardized prevalence rate of blindness and vision loss due to AMD in China is much higher than the average level of all other regions, while the age-standardized DALYs rate fell between that of upper middle-income regions and lower middle-income regions. This may be due to economic and infrastructure development, as well as the growing use of anti-VEGF drugs in China in recent years [23].

As the data in this study is from GBD 2019 study, the limitations of this study including the data are basely statistical assumption [8], as varying measurements were used for AMD assessment across studies. Furthermore, the lack of data from all of China’s provinces led to an inability to analyze the AMD burden across China’s provinces, which would have provided information for assessing the influence of economic development and geography on AMD disease burden.

The strength of the study is the use of large databases despite deficiency of available data in certain areas. Notwithstanding the limitations, this study offers health care policy-makers important evidence of the substantial and overall rising burden of disease associated with AMD.

## CONCLUSIONS

In conclusion, the prevalence of blindness and vision loss due to AMD and disease burden increased significantly in China from 1990 to 2019, especially for women and middle-aged and elderly population. Although the current eye health care policy in China could alleviate the AMD disease burden slightly, far more efficient treatments and policies are warranted. It is our hope that this study provides data to support and advocate for progress in more targeted and optimized health policies in the future.
